# Impact of cytomegalovirus exposure on disease severity, bacterial burden, immune responses and treatment outcomes in tuberculosis

**DOI:** 10.3389/fimmu.2025.1612709

**Published:** 2025-09-18

**Authors:** Bindu Dasan, Saravanan Munisankar, Nathella Pavan Kumar, Kadar Moideen, Arul Nancy Pandiarajan, Sujatha Nott, Vijay Viswanathan, Sivakumar Shanmugam, Syed Hissar, Kannan Thiruvengadam, Hardy Kornfeld, Subash Babu

**Affiliations:** ^1^ National Institutes of Health-NIAID-International Center for Excellence in Research, Chennai, India; ^2^ ICMR-National Institute for Research in Tuberculosis, Chennai, India; ^3^ Infectious Diseases, Dignity Health, Chandler, AZ, United States; ^4^ Prof. M. Viswanathan Diabetes Research Center, Chennai, India; ^5^ ICMR-Regional Medical Research Center, Port Blair, India; ^6^ UMass Chan Medical School, Worcester, MA, United States; ^7^ Laboratory of Parasitic Diseases, National Institutes of Allergy and Infectious Diseases, National Institutes of Health, Bethesda, MD, United States

**Keywords:** cytomegalovirus, tuberculosis, cytokines, disease severity, treatment outcomes

## Abstract

**Introduction:**

Tuberculosis (TB) continues to be one of the leading causes of global mortality. Recent evidence highlights human cytomegalovirus (CMV) as a risk factor for TB. However, the impact of CMV exposure on disease severity, bacterial burden, and TB treatment outcomes remain poorly understood.

**Methods:**

Serostatus of CMV was determined by assaying IgG titers in plasma samples by ELISA. Chest X-rays were employed to assess bilateral lung lesions and cavitary diseases, and sputum smear grades to measure bacterial loads in TB subjects. Treatment outcomes were defined as favorable or unfavorable. Cytokine profiles were measured using multiplex ELISA.

**Results:**

The study revealed that TB patients with CMV seopositivity had significantly higher bacterial loads (adjusted PR [aPR], 4·14; 95% CI, 2·21-7·16; p<0·001), bilateral lung lesions (aPR, 2·97; 95% CI, 1·71-5·17; P<0·001), cavitary lung lesions (aPR, 4·21; 95% CI, 1·98-6·24; p<0·001) and unfavorable treatment outcomes (aPR, 1·48; 95% CI, 1·08-2·69; p=0·05). Our data also show that TB is associated with significantly lower levels of IFNγ, IL-2, TNFα, IL-1α, and IL-1β but significantly higher levels of IL-10, IFNα, IFNβ, G-CSF, and VEGF in CMV exposed individuals compared to CMV non exposed individuals.

**Conclusion:**

Our findings reveal that CMV exposure worsens the severity of TB, increases bacterial burden, and leads to poorer treatment outcomes. The modulation of cytokine responses in TB patients with CMV exposure suggests a potential mechanism by which CMV may exacerbate TB pathogenesis.

## Introduction

Tuberculosis (TB) continues to pose a significant threat to global health, accounting for 10·6 million active cases and 1·7 million deaths annually (1). In addition, around one quarter of people worldwide carry *Mycobacterium tuberculosis* (Mtb) in a latent form that has the potential to transition into active TB, for instance, from coinfections (2). Chronic viral infections such as HIV, hepatitis C, and human T-lymphotropic virus type 1 increase the risk of TB disease (3–5). Similarly, recent studies suggest that human cytomegalovirus (CMV) infection increases the risk of progression to TB disease (6).

CMV, also known as human herpesvirus 5, is a member of the herpesviridae family (7). CMV infection is nearly ubiquitous, and it is prevalent in areas with high TB burdens. The epidemiology of the two illnesses is comparable in terms of age and sex distribution, heterogeneity in geographical prevalence, and overlapping risk factors (8). Primary CMV infection in immune-competent hosts is usually asymptomatic (9), but CMV can establish lifelong persistent infection in the host through the latent state, which revert to productive infection under circumstances of immunosuppression (10). Infection, reinfection, and reactivation of CMV might have extensive implications that modulate the immune response to TB (11).

Mtb control depends on the host’s innate and adaptive immune responses, particularly Th1 cell-mediated immunity, which is crucial for suppressing Mtb within macrophages in the lungs (12). Th1 cytokines like IFN-γ activate macrophages and cytolytic T cells to eliminate Mtb (13), while Th2 cytokines such as IL-4 and IL-13 hinder this process by promoting anti-inflammatory reactions (14). Although pro-inflammatory cytokines play a critical role in the immune response to TB (15), the impact of CMV exposure on cytokine responses in TB have not been explored.

To address this knowledge gap, our study aimed to compare disease severity, bacterial burden, and treatment outcomes in TB patients with or without CMV exposure. Moreover, to explore the immunological underpinnings of the interaction between CMV and TB, we examined the circulating plasma levels of a large panel of cytokines and pro-fibrotic factors in TB patients with or without CMV exposure.

## Materials and methods

### Ethics statement

The study was approved by the ethics committees of the National Institute for Research in Tuberculosis (NIRT) and the Prof. M. Viswanathan Diabetes Research Center (MVDRC) (ECR/51/INST/TN/2013/MVDRC/01).

### Patient consent statement

Informed written consent was obtained from all participants, and study procedures adhered to institutional ethical guidelines.

### Study population and data variables

Participants were recruited from Chennai, South India, as part of the prospective Effect of Diabetes on Tuberculosis Severity (EDOTS) study conducted from February 2014 to August 2018. The study included adult individuals aged 25 to 73 who were newly diagnosed with positive sputum smears and culture. Exclusion criteria were previous TB episodes, prior TB treatment, drug-resistant TB, positive HIV status, use of immunosuppressive medications, pregnancy, and lactation. Anthropometric measurements (height, and weight), and biochemical parameters were procured using standardized techniques. A complete blood count was done on all samples in a DxH 520 hematology analyzer (Beckman Coulter). Low body mass index (LBMI) was described based on the American Heart Association/American College of Cardiology guidelines (LBMI ≤ 18.5 kg/m^2^), overweight by body mass index (BMI) 25-29.9 kg/m^2^, and obesity defined by BMI threshold of ≥30.0 kg/m^2^. Diabetes was defined as glycated hemoglobin (HbA1c) reading of 6.5% or greater and a fasting blood glucose of ≥126 mg/dl, according to the American Diabetes Association criteria. Chest X-rays were utilized to assess the presence of bilateral lung disease and cavitary lesions and chest x-rays were read by 2 independent radiologists. Sputum smear grades were used to measure bacterial loads in individuals with TB and classified as 0, 1+, 2+, and 3+ with 0 being no bacteria in microscopy and 3+ the highest number of bacteria. The laboratory investigators were blinded to the chest x-ray and bacteriology results. All recruited TB patients received anti-TB treatment through Directly Observed Treatment Short Course (DOTS) therapy as per WHO recommendations, monitored by the National Tuberculosis Elimination Program (NTEP). Follow-up extended through 6 months of treatment and 1-year post-treatment completion. Treatment outcomes were defined as favorable or unfavorable. Favorable treatment outcome (cure) was defined as negative results of sputum cultures at months 5 and 6 of treatment without recurrent disease during follow-up. Unfavorable treatment outcomes included treatment failure defined as positive sputum culture results at month 5 or 6, all-cause mortality, or recurrent TB within 12 months after initial cure. These participants did not receive any treatment for CMV. Serostatus of CMV was determined by assaying the titer values of IgG in plasma samples using ELISA kit (MyBiosource) by following manufacturer’s instructions. Index values of <0·90 were considered negative, 0·90 to 1·1 were considered intermediate, and >1·1 were considered positive.

### Multiplex assays

Circulating plasma cytokines and pro-fibrotic levels were measured using multiplex Luminex assay (Magpix platform) (Bio-Rad Laboratories, Inc.). The analytes measured included cytokines (Interferon (IFN)-γ, Interleukin (IL)-2, Tumor Necrosis Factor (TNF-α), IL-4, IL-5, IL-6, IL-13, IL-3, IL-7, IFN-α, IFN-β, IL-1α, IL-1β, IL-1Ra, IL-17, Granulocyte colony-stimulating factor (G-CSF), Granulocyte-macrophage colony-stimulating factor (GM-CSF), IL-12, IL-15, IL-10, IL-25, IL-33) and pro-fibrotic factors(Vascular endothelial growth factor (VEGF), Epidermal growth factor (EGF), Platelet-derived growth factor (PDGF)-AB, PDGF-AB/BB, and Granzyme B). The experiment was conducted according to the manufacturer’s instructions (R&D Systems).

### Statistical analysis

Before analysis, the data was thoroughly checked for completeness and consistency. Continuous variables were examined for normality using the Shapiro-Wilks test and were found not to be normal. The data was then presented using frequency, percentages, median and quartiles. Geometric means (GM) were used for measurements of central tendency. Statistically significant differences between the three groups were analyzed by means of Kruskal-Wallis test with Dunn’s *post-hoc* for multiple comparisons. Mann–Whitney *U*-test with Holm’s correction for multiple comparisons was used between two groups. Differences in continuous variables between the groups were examined using the Wilcoxon rank sum test, while the relationship between groups and factors such as sputum smear grade, bilateral lung lesion, cavitary lesion, and TB treatment failure and relapse were examined using the Pearson chi-square test. Generalized linear models with binomial regression and log-link functions were used to identify key factors. The selection of covariates for the regression model was determined based on data availability, a review of relevant literature, and the opinions of subject matter experts. Prevalence ratios (PR) and adjusted prevalence ratios (aPR) were calculated along with the corresponding 95% confidence intervals (CIs). Covariates with significant PR, were considered when adjusting for aPR. Data analysis was performed using STATA software, version 15.0 (StataCorp., Texas, USA), with all P values considered two-sided and statistical significance set at the 0.05 α level.

## Results

### Study population characteristics

The total study population was 422 individuals with PTB, including 172 CMV positives, 108 CMV intermediates, and 142 CMV negatives. The median age was 44·0 (interquartile range [IQR], 36·0–51·5) years for CMV positive, 45·0 (IQR, 36·0–52·0) years for CMV intermediate and 45·0 (IQR, 36·0–52·0) years for CMV negative (*P*=0·003). There were no significant differences in gender, BMI, or alcohol use between the groups. However, significant differences were noted in age, smoking, HbA1c and anemia (**Table 1**). Individuals with CMV positive and CMV intermediate exhibited significantly lower levels of lymphocytes compared to CMV negative subjects (**Table 2**).

**Table 1:** Demographics and clinical characteristics of CMV seropositivity with TB individuals.

Variable	Overall, n=422^1^	CMV Positive n=172^1^	CMV Intermediate n=108^1^	CMV Negative n=142^1^	*p*-value*^2^*
Gender					
Female	182 (43.0)	72 (42.0)	46 (42.6)	64 (45.0)	0.84
Male	240 (57.0)	100 (58.0)	62 (57.4)	78 (55.0)	
Age in years, Median (IQR)	45.0 (36.0–52.0)	44.0 (36.0–51.5)	45.0 (36.0–52.0)	45.0 (36.0–52.0)	
18–34 (28)	104 (21.0)	30 (17.4)	25 (23.1)	49 (34.5)	0.003
35–44 (39)	111 (23.0)	51 (29.7)	30 (27.8)	30 (21.1)	
45–54 (49)	118 (35.0)	57 (33.1)	34 (31.5)	27 (19.0)	
≥55 (58)	89 (21.0)	34 (19.8)	19 (17.6)	36 (25.4)	
Smoking Status					
Non-smoker	182 (43.0)	86 (50.0)	27 (25.0)	69 (48.6)	0.02
Smoker	147 (35.0)	52 (30.0)	54 (50.0)	41 (28.9)	
Unknown	93 (22.0)	34 (20.0)	27 (25.0)	32 (22.5)	
Alcohol Status					
Alcohol use- Yes	179 (42.0)	78 (45.3)	45 (41.7)	56 (39.4)	0.61
Alcohol use - No	96 (23.0)	33 (19.2)	25 (23.1)	38 (26.8)	
Unknown	147 (35.0)	61 (35.5)	38 (35.2)	48 (33.8)	
BMI, (kg/m2), Median (IQR)	20.03 (17.50–23.30)	20.69 (18.22–23.00)	20.00 (17.42–23.37)	20.00 (17.42–23.37)	
Normal (18.5–24.9)	142 (33.6)	61 (35.4)	38 (35.2)	43 (30.3)	0.88
Underweight (<18.5)	189 (45.0)	72 (41.9)	48 (44.4)	69 (48.6)	
Overweight (25.0–29.9)	54 (13.0)	24 (14.0)	14 (13.0)	16 (11.3)	
Obesity (≥30.0)	37 (8.0)	15 (8.7)	8 (7.4)	14 (9.8)	
HbA1c %, Value, Median (IQR)	5.60 (4.80–10.40)	5.58 (4.28–10.95)	5.62 (4.55–10.95)	5.24 (4.50–10.30)	
NDM (<5.7)	234 (55.5)	84 (48.8)	55 (51.0)	95 (66.9)	0.05
PDM (>5.7–>6.2)	109 (25.8)	56 (32.6)	32 (29.6)	21 (14.8)	
DM (>6.2)	79 (18.7)	32 (18.6)	21 (19.4)	26 (18.3)	
Anemia (g/dL), Median (IQR)	12.40 (11.10–13.90)	11.65 (10.93–12.73)	11.65 (10.93–12.73)	12.60 (11.20–14.00)	
No-anemia	193 (45.7)	93 (46.0)	53 (49.0)	45 (43.0)	0.02
Anemia	229 (54.3)	95 (54.0)	55 (51.0)	78 (57.0)	

^1^Median (IQR) or Frequency (%).

^2^Wilcoxon rank sum test; Pearson’s Chi-squared test.

CMV, cytomegalovirus; TB, tuberculosis; BMI, Body mass index; HbA1c, Glycated hemoglobin; NDM, Non-diabetes mellitus; PDM, Pre-diabetes mellitus; DM, Diabetes mellitus.

**Table 2:** Hematological parameters of TB individuals with CMV seropositivity.

Parameters	CMV Positive GM (range)	CMV Intermediate GM (range)	CMV Negative GM (range)	p value
WBC count, x10^3^cells/ul	100.8 (63.0-178.0)	101.7 (51.0-197.0)	93.5 (62.0-157.0)	0.47
Lymphocyte count, x10^6^cells/ul	5961.8 (2000.0-9801.0)	6146.7 (1200.0-11760.0)	7378.6 (2964.0-14790.0)	0.002
Neutrophil count, cells/ul	7123.5 (2397.0-15000.0)	7434.7 (2964.0-15000.0)	6826.0 (2254.0-15136.0)	0.55
Monocyte count, cells/ul	694.5 (63.0-2509.0)	741.2 (224.0-1833.0)	660.8 (48.0-2394.0)	0.39
RBC, g/dL	4.7 (2.8-7.3)	4.7 (3.0-7.0)	4.8 (2.4-6.7)	0.13
Hb, g/dL	12.5 (7.2-18.3)	12.5 (6.3-19.5)	12.9 (7.7-20.1)	0.05
Hematocrit, %	38.3 (22.0-56.0)	33.7 (23.0-57.0)	39.3 (24.0-58.0)	0.06
Platelets, 10^3^/uL	372.7 (90.0-700.0)	407.8 (131.0-752.0)	390.5 (120.0-723.0)	0.11

CMV, cytomegalovirus; GM, geometric mean; WBC, white blood cell; RBC, red blood cell; Hb, hemoglobin.

### Association of clinical co-morbidities with CMV in TB individuals

No significant differences were observed in age, gender, BMI, alcoholism, or HbA1c between the two groups (**Table 3**). However, significant differences were noted in smoking and anemia. The PR for smoking individuals with CMV exposure was 1·57 (95% CI: 1·16–2·80; p = 0·02), and this association remained significant after adjusting for possible confounders (aPR, 1·40, 95% CI: 1·09–1·96; p = 0·04). The PR for anemia in individuals with CMV exposure was 2·69 (95% CI: 1·19–4·70; p = 0·01), and this association remained significant after adjusting for possible confounders (aPR 1·82, 95% CI: 1·09–3·86; p = 0·04).

**Table 3:** Association of clinical co-morbidities with CMV seropositivity with TB individuals.

Variable	CMV Positive PR (95% Cl)	*p*-value	CMV Positive aPR (95% Cl)	*p*-value
Socio-demographic characteristics-Sex				
Female	Reference	0.56	Reference	0.88
Male	0.87 (0.56–1.37)		0.935 (0.39–2.23)	
Age, years				
18–34	Reference	0.61	Reference	0.89
35–44	1.21 (0.64–2.68)		0.99 (0.40–2.43)	
45–54	0.87 (0.43–1.77)		0.78 (0.31–1.95)	
≥55	0.91 (0.41–2.05)		0.76 (0.27–2.11)	
BMI (kg/m^2^)				
Normal (18.5–24.9)	Reference	0.42	Reference	0.24
Underweight (<18.5)	1.71 (0.83–2.23)		1.5 (0.79–2.59)	
Overweight (25.0–29.9)	0.83 (0.42–1.64)		1.43 (0.61–3.31)	
Obesity (≥30.0)	0.64 (0.25–1.64)		1.39 (0.47–4.14)	
HbA1c (%)				
NDM (<5.7)	Reference	0.69	Reference	0.81
PDM (>5.7–<6.4)	1.02 (0.46–1.99)		0.84 (0.30–1.82)	
DM (>6.4)	0.97 (0.53–1.77)		0.75 (0.37–1.95)	
Anemia				
Male	Reference	0.01	Reference	0.04
Female (<12g/dL)	2.69 (1.19–4.70)		1.82 (1.09–3.86)	
Smoking	Reference	0.02	Reference	0.04
	1.57 (1.16–2.8)		1.4 (1.09–1.96)	
Alcoholism	Reference	0.61	Reference	0.85
	1.37 (0.71–2.57)		1.17 (0.69–2.32)	

CMV, cytomegalovirus; TB, tuberculosis; PR, prevalence ratio; aPR, adjusted prevalence ratio; CI, Confidence interval; BMI, Body mass index; HbA1c, Glycated hemoglobin; NDM, Non-diabetes mellitus; PDM, Pre-diabetes mellitus; DM, Diabetes mellitus.

### CMV seropositivity is associated with increased radiographic TB disease severity and greater bacterial burden

CMV seropositivity was significantly associated with an increased risk of cavitary disease (PR, 6·31; 95% CI, 3·12-10·42; p < 0·001) and bilateral lung lesions (PR, 5·24; 95% CI, 2·91-6·91; p< 0·001). After adjusting for confounding variables, CMV seropositivity remained significantly associated with a higher risk of cavitation (aPR, 4·21; 95% CI, 1·98-6·24; p < 0·001) and bilateral lung lesions, (aPR, 2·97; 95% CI, 1·71-5·17; p< 0·001) indicating increased TB disease severity in individuals with CMV exposure. Additionally, CMV seropositivity was significantly associated with an elevated risk of higher smear grades (PR, 6·31; 95% CI, 3·12-10·42; p< 0·001). This association persisted after adjusting for confounders, with CMV seropositivity remaining significantly associated with increased smear grades (aPR, 4·14; 95% CI, 2·21-7·16; p< 0·001), indicating higher bacterial burdens in TB patients with CMV exposure (**Table 4**).

**Table 4:** Association of CMV seropositivity with bacterial burden, disease severity and treatment failure/relapse in TB.

Outcome Variable	CMV/TB PR (95% Cl)	p-value	CMV/TB aPR (95% Cl)	p-value
Sputum smear grade	6.31 (3.12–10.42)	<0.001	4.14 (2.21–7.16)	<0.001
Bilateral lung lesions	5.24 (2.91-6.91)	<0.001	2.97 (1.71-5.17)	<0.001
Cavitary lung lesions	5.57 (3.16-9.05)	<0.001	4.21 (1.98-6.24)	<0.001
TB treatment failure/relapse	1.67 (1.26–2.95)	0.01	1.48 (1.08–2.69)	0.05

CMV, cytomegalovirus; TB, tuberculosis; PR, prevalence ratio; aPR, adjusted prevalence ratio; CI, confidence interval.

### CMV seropositivity is associated with increased risk of unfavorable TB treatment outcomes

CMV seropositivity was significantly associated with an increased risk of unfavorable treatment outcomes (PR, 1·67; 95% CI, 1·26–2·95; p = 0·01). This association persisted even after adjusting for confounding variables, with CMV remaining significantly associated with unfavorable treatment outcomes (aPR, 1·48; 95% CI, 1·08-2·69; p = 0·05). These findings indicate a heightened risk of treatment failure or TB recurrence in TB patients with CMV (**Table 4**).

### CMV seropositivity is associated with altered levels of cytokines and pro-fibrotic factors in TB

**Figure 1:**
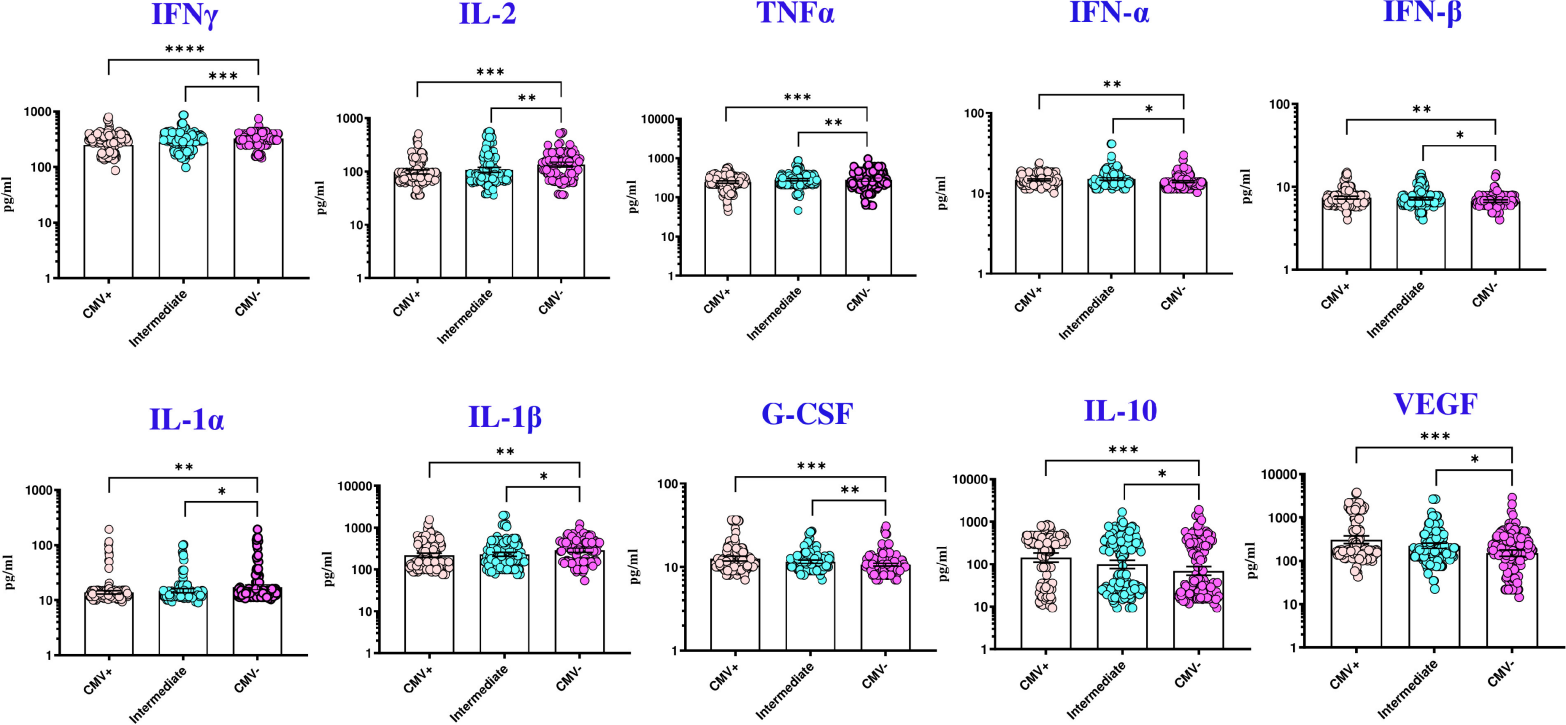
CMV seropositivity is associated with altered levels of cytokines in TB individuals. The figure illustrates the cytokine profile in CMV positive, intermediate and negative individuals with TB. Each data point represents an individual subject, with the bar indicating the geometric mean (GM) cytokine level. Statistical analysis was performed using the Kruskal-wallis test. *p<0.05, **p<0.005, ***p<0.0005, ****p<0.00005.

To explore a plausible mechanism by which CMV impacts TB individuals, we measured the plasma levels of various cytokines and pro-fibrotic factors in TB individuals with CMV positive, intermediate and negative groups. The circulating levels of IL-3, IL-7, IL-4, IL-5, IL-6, IL-1Ra, IL-17, GM-CSF, IL-12, IL-15, IL-25, IL-33, IL-13, EGF, PDGF-AA, PDGF-AB BB, and Granzyme B did not significantly differ between the groups. However, pro-inflammatory cytokines (IFN-α, IFN-β, G-CSF), regulatory cytokines (IL-10), and pro-fibrotic factors (VEGF) were significantly elevated in TB with CMV positive and CMV intermediate individuals compared to those without CMV. Conversely, the circulating plasma levels of type 1 cytokines (IFN-γ, TNF-α, IL-2), and proinflammatory cytokines (IL-1α, and IL-1β) were significantly diminished in TB with CMV positive and CMV intermediate individuals compared to those without CMV (**Table 5**, **Figure 1**).

**Table 5:** Association of CMV seropositivity with altered levels of cytokines in TB individuals.

Parameters	CMV Positive GM (range)	CMV Intermediate GM (range)	CMV Negative GM (range)	p value
IFNγ	250.1 (86.8-791.5)	284.8 (98.0-865.4)	311.5 (86.8-738.1)	<0.0001
IL-2	98.4 (36.0-509.3)	108.1 (36.0-571.1)	128.9 (36.5-544.6)	0.002
IL-6	113.1 (43.5-309.8)	117.4 (35.8-519.0)	162.7 (33.6-1925.6)	0.06
TNFα	280.2 (46.3-872.1)	277.0 (61.9-962.7)	244.0 (43.3-584.3)	0.0006
IFN-α	14.6 (10.0-24.0)	15.0 (11.3-41.3)	13.9 (10.1-30.0)	0.001
IFN-β	7.4 (4.0-15.0)	7.2 (4.0-14.4)	6.8 (4.0-15.0)	0.003
IL-3	15.1 (10.2-40.7)	15.0 (10.2-51.0)	14.4 (9.1-50.0)	0.05
IL-7	18.6 (3.2-83.4)	16.5 (3.2-79.5)	15.6 (3.9-88.3)	0.10
IL-1α	13.9 (9.3-193.8)	14.8 (9.0-193.8)	17.2 (10.3-193.8)	0.002
IL-1 β	218.9 (76.5-1551.0)	231.3 (76.5-1974.7)	285.1 (54.0-1217.6)	0.002
IL-1Ra	538.6 (39.2-8042.4)	410.8 (21.9-3957.9)	416.9 (26.6-8042.4)	0.05
IL-17	212.2 (151.1-452.0)	241.5 (110.0-480.6)	217.7 (140.6-542.0)	0.05
G-CSF	12.6 (7.0-36.7)	11.7 (7.0-36.7)	10.6 (7.0-31.0)	0.0002
GM-CSF	180.5 (58.6-1190.1)	187.9 (45.9-1190.1)	218.9 (37.2-1300.0)	0.09
IL-12	17.5 (5.2-57.8)	20.5 (5.2-180.1)	17.2 (4.0-180.1)	0.09
IL-15	13.4 (5.0-42.0)	13.7 (5.0-42.0)	13.2 (5.0-43.3)	0.68
IL-4	15.7 (10.0-38.2)	14.9 (10.0-50.0)	14.9 (10.0-39.3)	0.05
IL-5	14 (9.4-113.3)	12.4 (5.7-113.3)	12.9 (5.7-66.3)	0.05
IL-13	27.4 (10.7-189.2)	27.6 (5.2-189.2)	24.3 (5.2-146.8)	0.12
IL-10	143.6 (9.3-856.6)	98.7 (9.3-1693.3)	69.5 (9.3-1915.2)	0.0002
IL-25	17.1 (7.1-38.3)	20.6 (6-38.3)	18.1 (6.1-38.0)	0.07
IL-33	25.5 (13.6-185.0)	27.2 (10.0-145.0)	26.8 (10.0-185.0)	0.05
VEGF	307.9 (42.5-3789.9)	224.3 (22.5-2652.8)	150.5 (14.4-2906.1)	0.0004
EGF	256.0 (25.8-50876.8)	234.7 (5.8-27381.9)	410.0 (17.8-34799.5)	0.08
PDGF-AA	2446.3 (404.6-25266.4)	2976.3 (500.0-25607.1)	2177.1 (450.0-26045.3)	0.06
PDGF-AB BB	1574.2 (277.0-4840.0)	16361.7 (400.0-4532.4)	1442.7 (308.0-4993.0)	0.54
Granzyme B	18.2 (12.0-42.5)	17.3 (11.5-51.0)	17.9 (11.0-34.2)	0.10

CMV, cytomegalovirus; GM, geometric mean; IFN γ, Interferon gamma; IL-2, Interleukin-2; TNF-α, Tumor necrosis factor alpha; IFN α, Interferon alpha; IFN β, Interferon beta; IL-6, Interleukin-6; IL-3, Interleukin-3; IL-7, Interleukin-7; IL-1α, Interleukin-1α; IL-1β, Interleukin-1β; IL-1Ra, Interleukin-1Ra; IL-17, Interleukin-17; G-CSF, Granulocyte colony stimulating factor; GM-CSF, Granulocyte-macrophage colony stimulating factor; IL-12, Interleukin-12; IL-15, Interleukin-15; IL-4, Interleukin-4; IL-5, Interleukin-5; IL-13, Interleukin-13; IL-10, Interleukin-10; IL-25, Interleukin-25; IL-33, Interleukin-33; VEGF, Vascular endothelial growth factor; EGF, Epidermal growth factor; PDGF-AA, Platelet-derived growth factor; PDGF-AB BB, Platelet-derived growth factor.

## Discussion

TB management depends on understanding the predisposing factors associated with disease progression and poor anti-TB treatment outcomes (16). There is increasing evidence on the role of coinfections in TB pathogenesis, as chronic viral infections have been demonstrated to promote Mtb, delay Mtb-specific T-cell priming, and contribute to TB morbidity in experimental conditions (17). Among these, CMV infection has recently been implicated as a potential risk factor for TB disease (18). A large cross-sectional study from the U.S. National Health and Nutrition Examination Survey (NHANES) found that CMV seropositivity was independently associated with latent TB infection (LTBI), with higher CMV antibody levels correlating with increased LTBI prevalence, suggesting an immunological link between CMV-induced immune activation and TB susceptibility (19). However, despite growing evidence, only a few published studies have investigated the associations between the two infections (8, 20) and the mechanisms causing TB susceptibility are unknown. Our study is the first to explore the impact of CMV exposure on TB disease severity, bacterial burden, and treatment response.

We examined the impact of CMV exposure on clinical characteristics, disease severity, and treatment outcomes in a cohort of 422 PTB patients, stratified into CMV-positive, CMV-intermediate, and CMV-negative groups based on CMV serostatus. Our findings revealed that CMV-positive and CMV-intermediate individuals had significantly lower lymphocyte counts compared to CMV-negative individuals. Although no significant differences were observed in demographic characteristics such as age, gender, and BMI, the analysis identified smoking and anemia as clinical comorbidities significantly associated with CMV exposure. Adjusted prevalence ratios indicated that smoking and anemia were independently associated with CMV positivity in TB patients, highlighting the need to consider these factors when evaluating TB disease severity and outcomes in individuals exposed with CMV. This is consistent with recent findings suggesting the association of CMV serostatus in those with a heavy smoking history and anemia (21, 22).

In addition, our results showed that CMV exposure was significantly associated with increased radiographic TB disease severity, evidenced by a higher prevalence of cavitary disease and bilateral lung lesions in CMV-positive individuals compared to CMV-negative individuals. After adjusting for potential confounders, the prevalence of having cavitary disease and bilateral lung lesions remained markedly elevated in CMV-positive individuals. Furthermore, CMV positivity was associated with higher bacterial burden, as indicated by smear grades in these patients. The persistence of these associations after adjustment for confounders underscores the potential role of CMV exposure in exacerbating TB pathology, possibly through modulation of host immune responses, which may facilitate Mtb replication and dissemination. A recent meta-analysis of 15 studies involving 38,618 patients supports this, demonstrating that CMV infection is significantly associated with increased risk of active TB (OR: 3.20; 95% CI: 2.18–4.70), with a clear dose-response relationship between CMV antibody levels and TB risk (23). Further evidence from a recent longitudinal cohort study demonstrated that in both infant and adolescent cohorts, CMV-specific IFN-γ responses were associated with CD8+ T-cell activation and an increased risk of TB disease, as well as a shorter time to TB diagnosis, supporting the connection between CMV-induced immune activation and TB susceptibility (11). Moreover, a Phase 2b clinical trial of a developmental TB vaccine found that immune activation characterized by increased HLA-DR expression on CD4+ T cells was linked to a higher risk of TB disease in South African infants, underscoring how immune activation—potentially driven by CMV—may influence TB disease progression (24).

The association of CMV seropositivity with unfavorable TB treatment outcomes is particularly concerning as it was significantly linked to a higher risk of treatment failure or TB recurrence, even after adjusting for confounders. CMV presumably contributes to poor treatment responses in TB patients through persistent effects on protective immune functions. Given the high seroprevalence of CMV in the Indian population (25) and our findings presented here, consideration should be given to screening for CMV antibodies in TB patients as a marker for disease severity and a predictor of treatment outcomes.

Biomarkers for disease severity and unfavorable TB treatment outcomes can play a major role in identifying novel TB intervention strategies (26–32). Since cytokines are critical in the host defense against mycobacterial infections and serve as markers of disease severity and bacterial burden in active TB (33, 34), we explored cytokine responses that reflect cell-mediated immunity in TB infection. CMV is known to imbalance systemic cytokine, T-cell, and macrophage responses and inflammation (35). Our findings reveal that TB with CMV seropositivity is associated with reduced levels of protective cytokines, altered pro-inflammatory cytokines and elevated pro-fibrotic factors, potentially aggravating TB pathogenesis.

Studies in human observational cohorts and animal models have established that Th1 cytokines, such as IFN-γ, TNF-α, and IL-12, are crucial for preventing TB infection and disease progression (36, 37). TNF-α, in particular, is essential for bacterial control during Mtb infection through its activation of phagocytes and granuloma formation (38). Our findings indicate that CMV-seropositive TB patients exhibit lower circulating Th1 cytokine levels, suggesting an immunological mechanism driving worse TB severity (39). Additionally, decreased IL-1 levels in CMV-positive individuals align with previous reports of immune suppression in TB-HIV coinfection (40). Type I interferons (IFN-α and IFN-β), which are protective against viral infections (41), may have detrimental effects in TB (42). We observed elevated Type I IFNs in CMV-positive individuals, consistent with reports linking heightened Type I IFNs to adverse TB outcomes in both mouse models and humans (43, 44). Chronic viral infections, including CMV, can also enhance the production of immunosuppressive factors like IL-10, which lowers immune protection against Mtb and promotes Mtb persistence (45). Emerging evidence suggests that CMV may impair local immune responses in the lung through immunosuppressive mechanisms, including viral IL-10–mediated polarization of monocytes toward an anti-inflammatory macrophage phenotype, potentially compromising Mtb control at the site of infection (46). Blocking IL-10 has been shown to stabilize bacterial load and improve survival in Mtb-infected mice (47). Our data support these findings, showing elevated IL-10 levels in CMV-positive individuals, which could exacerbate TB infection, as observed in transgenic mouse models (48). Furthermore, we noted increased vascular endothelial growth factor (VEGF) levels in CMV-positive individuals, a biomarker associated with TB disease severity and bacterial burdens (49), corroborating earlier studies on systemic VEGF elevation in TB patients with extensive disease involvement (50).

In this study, rigorous control was applied to several variables, including age, gender, BMI, diabetes, smoking status, and alcohol use, which are known to influence bacterial burdens and disease severity. Because smoking status and anemia differed between the CMV-positive and CMV-negative groups, we adjusted for their effects and demonstrated that the significant association of CMV exposure with TB remains despite this difference. Therefore, our study demonstrates an important association between CMV seropositivity and the increased risk of cavitation and bilateral lung lesions, even under rigorous screening criteria. These results corroborate other studies that have suggested that these lesions have a detrimental effect on patients and may result in poor treatment outcomes, relapses, and drug resistance (51). As estimated by sputum smear grades in PTB, our data confirm an important association of CMV seropositive status with mycobacterial burdens, a key indicator of transmission risk and disease severity (52). Our data further confirms that TB individuals with CMV exposure were at a significantly higher risk of experiencing unfavorable treatment outcomes, including treatment failure or TB recurrence. This finding aligns with previous research indicating that patients with TB-HIV coinfection exhibit poor treatment outcomes and a heightened degree of inflammatory perturbation (53).

Our study suffers from the limitation of relying only on IgG positivity as a diagnostic for CMV exposure. The results provide a strong rationale to measure viral load, but that it not possible with the available samples and will require a new prospective study. Another limitation of our study is that cytokine levels exhibit a great degree of overlap between groups and that there is variability in the responses of different individuals in the same group. It is possible that other factors not examined in this study could have contributed to the differential responses. This variability could limit to use of cytokine levels as predictive biomarkers, but does not diminish their value, with statistical adjustment, as informative markers of underlying biological processes.

In conclusion, this study highlights the complex interplay between CMV and TB disease, suggesting that CMV exposure contributes to increased TB disease severity, greater bacterial burdens, and a higher risk of unfavorable treatment outcomes. Future studies should focus on elucidating the mechanisms underlying CMV-mediated alterations in TB immunopathogenesis and exploring potential therapeutic interventions.

## Data Availability

The original contributions presented in the study are included in the article/supplementary material, further inquiries can be directed to the corresponding author/s.
